# Supplementary material for: DNA Damage Regulates Senescence-Associated Extracellular Vesicle Release via the Ceramide Pathway to Prevent Excessive Inflammatory Responses
Source: Int J Mol Sci. 2020 May 25;21(10):3720. doi: 10.3390/ijms21103720 (PMC7279173; doi:10.3390/ijms21103720)
Supplement: Supplementary file 1 [file ijms-21-03720-s001.pdf]

Supplementary information for

DNA damage regulates senescence-associated extracellular vesicle release via the ceramide pathway to prevent excessive inflammatory responses.

Kazuhiro Hitomi, Ryo Okada, Tze Mun Loo, Kenichi Miyata, Asako J. Nakamura and Akiko Takahashi*

* Corresponding author: akiko.takahashi@jfcr.or.jp

This supplementary information file includes:

Supplementary Figure 1 to 4

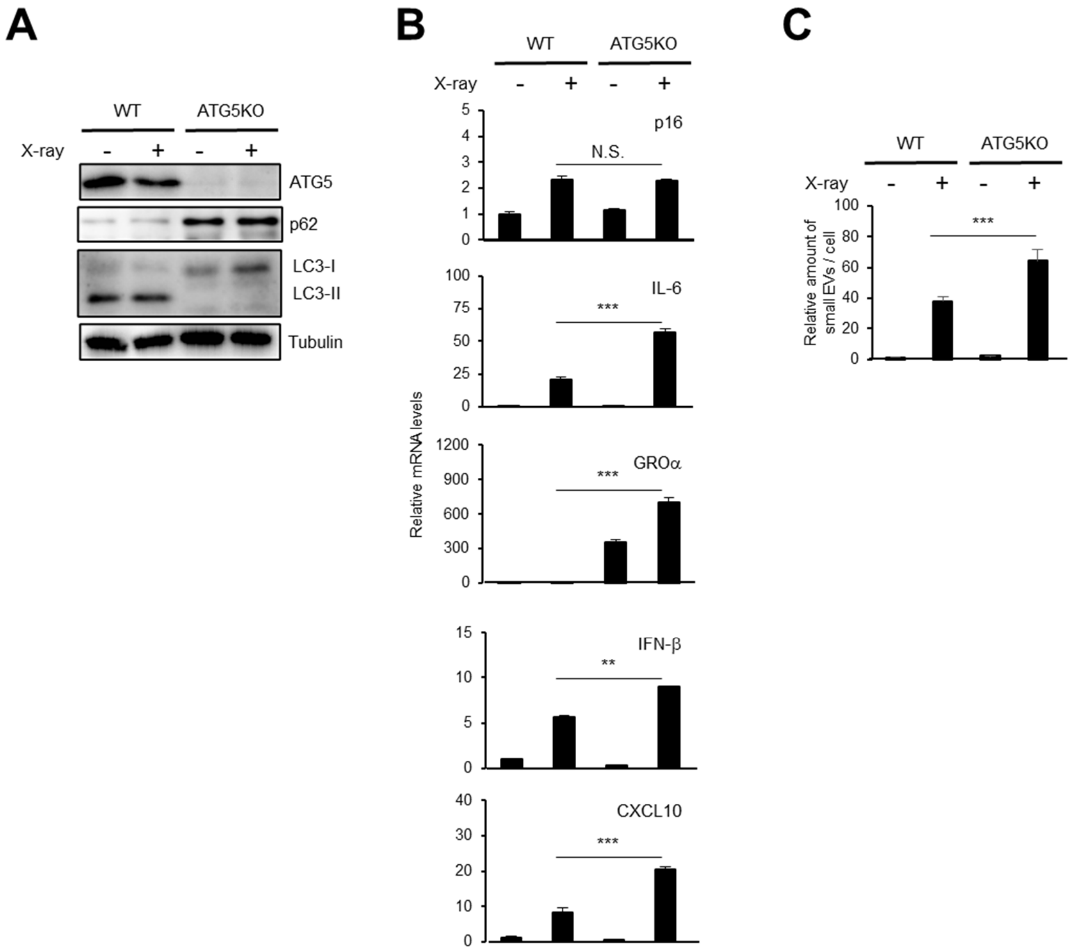

Figure S1. DNA damage enhanced small EV release from *ATG5* knockout mouse embryonic fibroblasts (MEFs) compared with their release in wild-type MEFs. **(A–C)** Murine primary MEFs derived from wild type (WT) or *ATG5*$^{-/-}$ mice were rendered senescent by 20Gy ionizing radiation (IR). After 10 days, these cells were subjected to western blotting using antibodies shown right (**A**), RT-qPCR analysis of SASP-factor gene expression (**B**) or to NanoSight analysis (NTA) for quantitative measurement of isolated small EV particles (**C**). For all graphs, error bars indicate mean ±standard

deviation (s.d.) of triplicate measurements. *P* values was calculated by unpaired two-tailed Student's *t*-test (***P* < 0.01, ****P* < 0.001, N.S. (not significant)).

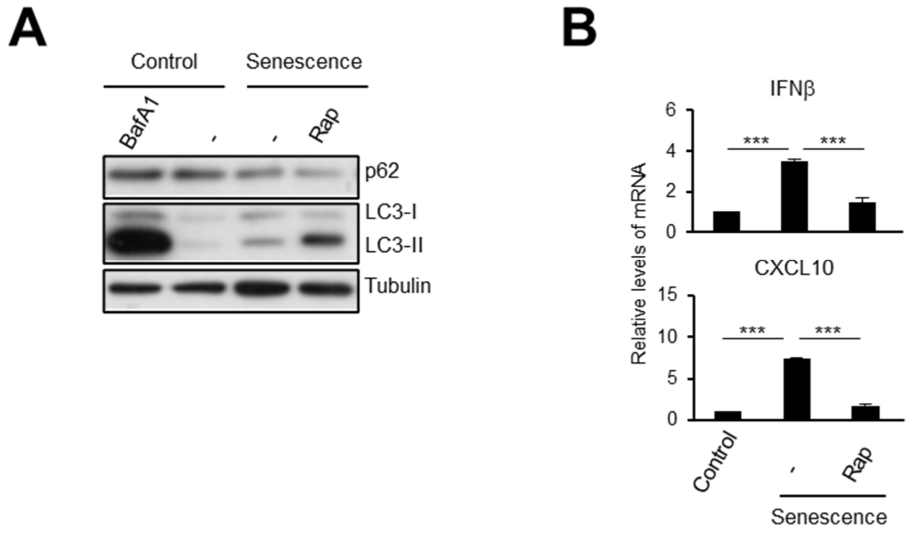

Figure S2. Activation of the autophagy pathway prevents inflammatory gene expression in senescent cells. **(A,B)** Pre-senescent TIG-3 cells were rendered senescent by ectopic expression of oncogenic *ras* (+HRasV12). These cells were treated with 50 ◎M bafilomycin A1 (BafA1) or 10 ◎M rapamycin (Rap) for 48 h and then subjected to western blotting using antibodies shown right (**A**) or RT-qPCR analysis of IFN-◎ and CXCL10 gene expression (**B**). For all graphs, error bars indicate mean ± standard deviation (s.d.) of triplicate measurements. *P* values was calculated by unpaired two-tailed Student's *t*-test (****P* < 0.001).

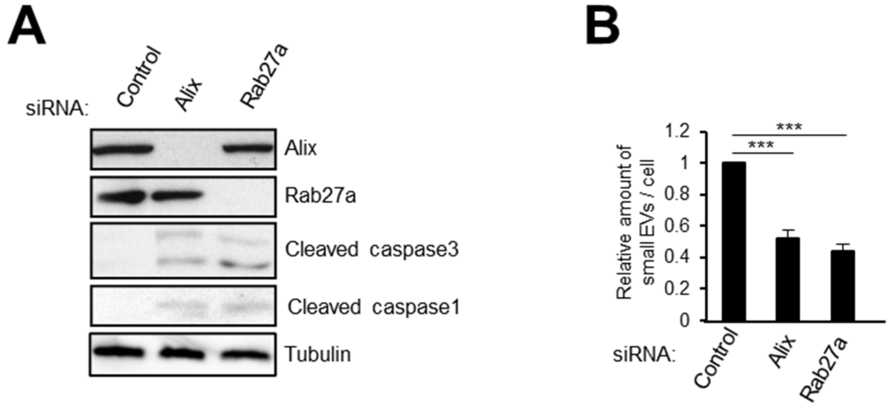

Figure S3. Inhibiting the small EV pathway provokes inflammasome activation and apoptosis in Bacillus Calmette–Guérin (BCG)-infected cells. (**A, B**) THP-1 cells were treated with 100nM PMA for 7 days and transfected with indicated siRNA oligos followed by infection with Bacillus Calmette–Guérin (BCG). These cells were subjected to western blotting using antibodies shown right (**A**) and to NanoSight analysis (NTA) for quantitative measurement of isolated small EV particles (**B**). For all graphs, error bars indicate mean ± standard deviation (s.d.) of triplicate measurements. *P* values was calculated by unpaired two-tailed Student's *t*-test (****P* < 0.001).

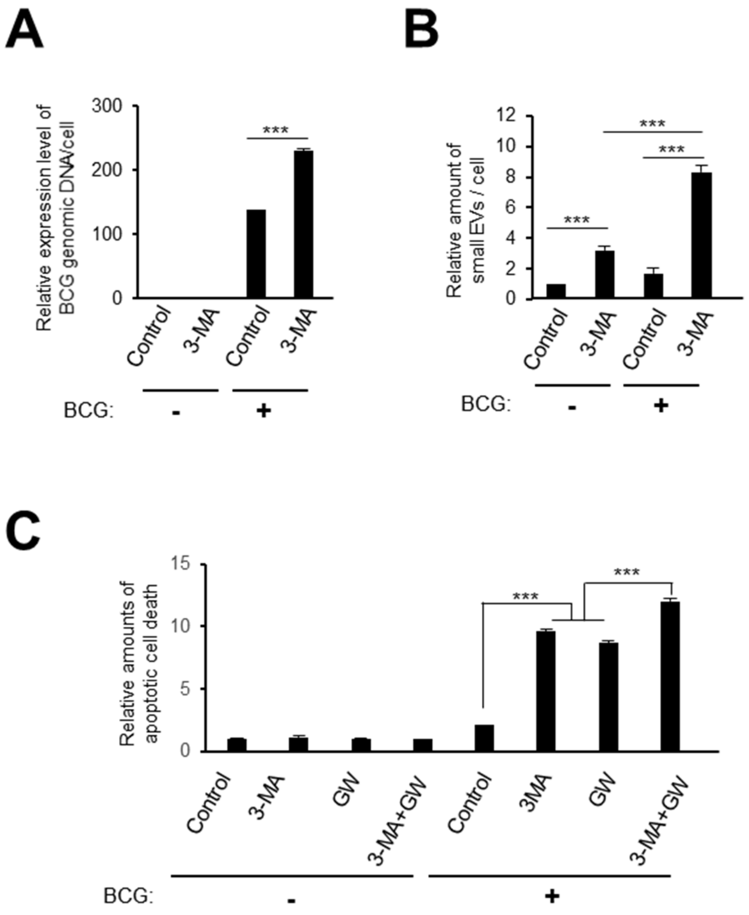

Figure S4. Inhibiting both autophagy and small EV biogenesis promotes apoptotic cell death following Bacillus Calmette-Guérin (BCG) infection. (**A**–**C**) THP-1 cells were treated with 100 nM PMA for 7 days and followed by infection with Bacillus Calmette–Guérin (BCG) with or without 5 mM 3-MA for 9 h. These cells were subjected to quantitative measurement of isolated bacterial DNA from cells using qPCR (**A**), NanoSight analysis (NTA) for quantitative measurement of isolated small EV particles (**B**) or to apoptotic cell death analysis (**C**). For all graphs, error bars indicate mean ± standard deviation (s.d.) of triplicate measurements. *P* values was calculated by unpaired two-tailed Student's *t*-test (***$P < 0.001$).
